# Why antimicrobial resistance messaging fails: qualitative insights interpreted through the elaboration likelihood model

**DOI:** 10.1093/jacamr/dlaf148

**Published:** 2025-08-08

**Authors:** Eva M Krockow, David R Jenkins, Samkele Mkumbuzi, Stephen J Flusberg, Carolyn Tarrant

**Affiliations:** School of Psychology and Vision Sciences, University of Leicester, University Road, Leicester LE1 7RH, UK; Leicester Royal Infirmary, University Hospitals of Leicester NHS Trust, Leicester LE1 5WW, UK; Department of Medicine, Division of Infectious Diseases & HIV Medicine, Groote Schuur Hospital, University of Cape Town, Cape Town, South Africa; Department of Cognitive Science, Vassar College, Poughkeepsie, NY, USA; School of Medical Sciences, University of Leicester, Leicester, UK

## Abstract

**Background:**

Antimicrobial resistance (AMR) is a global threat, yet public awareness remains low. This study examined perceptions of current AMR communications to improve knowledge, extending previous research through qualitative data analysed using the Elaboration Likelihood Model (ELM).

**Methods:**

We held 3 focus groups (*n* = 15) with UK patients with recent experience of AMR and 4 (*n* = 14) with hospital doctors experienced in AMR treatment and communication. Semi-structured questions explored perceptions of public AMR messaging. Data were analysed using reflexive thematic analysis.

**Results:**

Most participants found public AMR information difficult to access, overly technical, and unclear. They struggled to find personal and cultural relevance, described the tone as punitive and highlighted contradictory advice (e.g. discouraging antibiotic use while recommending full course completion), undermining argument quality. Some appreciated buzzwords like ‘superbugs’, but most felt that messages lacked impact and ‘punch’. When viewed through the ELM, the problematic tone and lack of personalisation reduced recipients’ motivation. The lack of readily available, clear information hindered their ability to engage deeply with messages via ‘central route’ processing, reducing the likelihood of elaboration and subsequent persuasion. Attitude change from peripheral route information processing was equally questionable given the lack of persuasive message cues.

**Conclusions:**

Current AMR messaging is insufficient and communication theory could highlight areas for improvement. Our ELM analysis suggests a need to enhance motivation, capability, and argument quality while adding persuasive, peripheral cues. Personally and culturally tailored messages with a positive, solution-focused tone and simplified, engaging language may boost impact and promote lasting attitude change.

## Introduction

Antimicrobial resistance (AMR) is widely recognized as one of the biggest threats to modern medicine. In 2019, bacterial AMR was associated with 4.95 million deaths globally,^1^ and projections indicate that AMR-related deaths could rise to 10 million per year by 2050.^2^ In spite of these worrying forecasts, public awareness remains low.^3,4^ Research shows people underestimate the risks of AMR or hold misconceptions, such as believing that antibiotics are effective against viral illnesses or that the body becomes resistant to antibiotics, rather than the bacteria.^4,5^ Ignorance and misbeliefs can give rise to inappropriate behaviours, exacerbating the problem. These include inappropriate demands for antibiotics, sharing antibiotics and failing to follow treatment recommendations.^3^

Effective risk communication about AMR is essential to increase awareness, dispel myths, optimize behaviours, and garner public support for financial investment into the antibiotic treatment pipeline. It is also critical for the development of new diagnostics and policies to curb antibiotic overuse. While public AMR messaging spans news, social media, and health campaigns, initial research suggests that existing messages fail to achieve the desired impact.

Media reporting about AMR has been criticized for its superficial, attention-grabbing narratives,^6^ which often lack recommendations about risk reduction measures.^7^ Compared with the related topic of sepsis, AMR messages rarely incorporate patient stories to enhance personal relevance.^8,9^ The frequent use of hyperbolic, apocalyptic imagery may be counterproductive, leading to de-sensitisation and apathy,^10,11^ and overly simplistic war narratives (e.g. calling to fight evil bacteria) may misrepresent the nuanced reality of human-bacteria relationships.^12–14^

Several reviews aimed to evaluate the effectiveness of public education campaigns. Although some campaigns showed measurable success.^15^ many failed to sustain behaviour change.^16^ The most successful campaigns used disease-specific messages targeted to selected socio-demographic groups, simple materials that aided recall, multiple media channels and interactive elements such as pledges.

Some research used experimental approaches and hypothetical vignettes to test the effectiveness of specific AMR message elements. Simply explaining that antibiotics are ineffective against viral illnesses reduced antibiotic expectations.^17^ Additionally, information designed to promote empathy^18^ or fear^19,20^ may decrease patient demands for antibacterials, whereas labelling AMR as a ‘silent’ pandemic could dampen risk perceptions.^21^ A mixed-methods evaluation of AMR message framing highlighted many problematic aspects of current AMR narratives,^22^ including inconsistent use of language and a lack of personal relevance for present-day decision-making. Follow-up experiments reinforced this, showing that inconsistent, abstract terms like ‘AMR’ and ‘antimicrobial resistance’ were ineffective in public communications.^23,24^

While past research has offered useful insights into AMR messaging, few studies use in-depth qualitative methods or apply broader risk communication theories. Indeed, the lack of theoretical foundations was explicitly stated as a limitation in educational campaigns.^16^ Much of the discourse around AMR messaging remained narrowly focused on the topic itself, including specific framing recommendations like portraying AMR as ‘undermining modern medicine’.^22^ This overlooks the opportunity to apply broader risk communication theory to AMR.

The Elaboration Likelihood Model (ELM)^25^ is a key theory in risk communication, proposing that message impact depends on how deeply it’s processed. When people are motivated and able, they engage centrally, leading to lasting attitude change. With low motivation or ability, they rely on peripheral cues, resulting in more temporary shifts. Introduced in the 1980s, the ELM remains central to persuasion theory.^26^ It continues to be used to predict persuasiveness of health messages, for example, about pandemic behaviours^27^ and vaccination.^28^

This study builds on previous AMR communications research^16,22,23^ by collecting qualitative data and providing theory-driven interpretations and recommendations, using the ELM. Our approach was guided by the following research questions:

1. How do patients with lived experiences of antibiotic resistant infections and frontline healthcare staff involved in antibiotic prescribing perceive current public AMR messages and terminology?
2. What are these individuals’ insights and opinions on public communication needs and successful messages about AMR?
3. What insights and recommendations can be drawn from mapping findings against relevant theoretical frameworks, notably the ELM?

## Methods

### Design

We conducted semi-structured focus groups with members of the UK general public, who had recent (past ≤ 5 years) experience of infections caused by multidrug-resistant bacteria, and secondary care doctors, who had professional experiences of treating multidrug-resistant infections.

### Participants

We recruited an opportunity sample of 15 patients via: (1) personal invitation letters sent to individuals identified via hospital records at University Hospital of Leicester NHS trust and (2) social media adverts on X, Bluesky, LinkedIn and Facebook. Individuals identified via hospital records were identified on the basis of the resistance of their isolates, prioritising the isolates with greatest resistance (e.g. carbapenem resistance), followed by isolates with less extent of resistance (e.g. extended spectrum beta lactamase producers). The response rate was approximately 7.5%. These 15 patient participants were allocated to two virtual focus groups and one face-to-face focus group at the University of Leicester, with group sizes ranging between 4 and 6. Additionally, we recruited an opportunity sample of 14 hospital doctors with experience of treating drug-resistant infections via: (1) emails sent to existing professional contacts (2) social media adverts on X, Bluesky, and LinkedIn. These 14 participants were allocated to 4 virtual focus groups, with group sizes ranging between 3 and 4. For demographic data see Table 1.

**Table 1.** Aggregate demographic data of patient and doctor focus group participants

	Patient participants (*n* = 15)	Doctor participants (*n* = 14)
Gender	Male = 5	Male = 8
	Female = 10	Female = 6
Age	Mean = 46.5 years	Mean = 43.2 years
	SD = 19.85	SD = 13.5
	Range = 21–74 years	Range = 23–64 years
	Prefer not to say = 1	Prefer not to say = 1
Ethnicity	Asian or Asian British = 5	White = 8
	Black, African, Caribbean or Black	Asian or Asian British = 4
	British = 5	Black, African, Caribbean or Black
	White = 4	British = 1
	Prefer not to say = 1	Other (Arab) = 1
Education level (patients)/Career stage (doctors)	Highschool graduate or equivalent = 1	Consultant^a^ = 8
	College sixth form graduate or equivalent = 3	Foundation years doctor^b^ = 4
	University (bachelors) degree graduate or equivalent = 8	Registrar^c^ = 2
	Postgraduate degree graduate = 2	
	Prefer not to say = 1	
Geographic region	England/London: 5	England/East Midlands: 13
	England/East Midlands: 4	England/London: 1
	England/West Midlands: 1	
	England/North West: 1	
	England/South East: 1	
	England/Yorkshire & the Humber: 1	
	Wales/Cardiff: 1	
	Northern Ireland/Belfast: 1	

^a^Consultant refers to a senior hospital doctor equivalent to an attending physician in the U.S. or a specialist doctor in other healthcare systems.

^b^Foundation year doctor refers to a junior doctor undergoing the first 2 years of post-medical school training.

^c^Registrar refers to a doctor undergoing specialist training.

### Materials

Materials included a demographic questionnaire and semi-structured topic guides for patients and doctors, available in the Supplementary Materials (available as Supplementary data at *JAC-AMR* Online). Topic guides explored awareness and perceptions of existing AMR communication materials, including prompts regarding the usefulness of current terminology.

### Procedure

Patient focus groups were held from October to November 2024, and doctor focus groups from December 2024 to February 2025. All participants provided informed consent. Sessions were audio-recorded, transcribed verbatim and de-identified before analysis. Patient focus group discussions lasted 1.5 h within a 3-h research session, while doctor discussions lasted 30 min within a 1-h session. The second half of each session focused on co-designing new AMR risk messages (reported elsewhere).

### Data analysis

Analysis was informed by a critical realist approach,^29,30^ which recognizes the importance of socio-cultural mediators in shaping understandings of reality. Data were uploaded to NVivo software and analysed by the lead researcher using reflexive thematic analysis.^31,32^ We followed the six recommended steps: (1) initial familiarisation with the transcripts, (2) open coding that resulted in a largely descriptive coding framework, (3) grouping of initial codes within larger themes, (4) iterative review of themes to check the fit, (5) definition and description of final themes and (6) subsequent write-up. Stages 3 and 4 were supported by visual mind-mapping to group codes into meaningful categories and through discussions with the project team. The lead researcher actively reflected on potential bias. This included a preconception of AMR risk communication being inadequate, which was based on previous academic literature. The researcher kept a coding book with reflexive notes to minimize bias.

## Results

**Figure 1.**
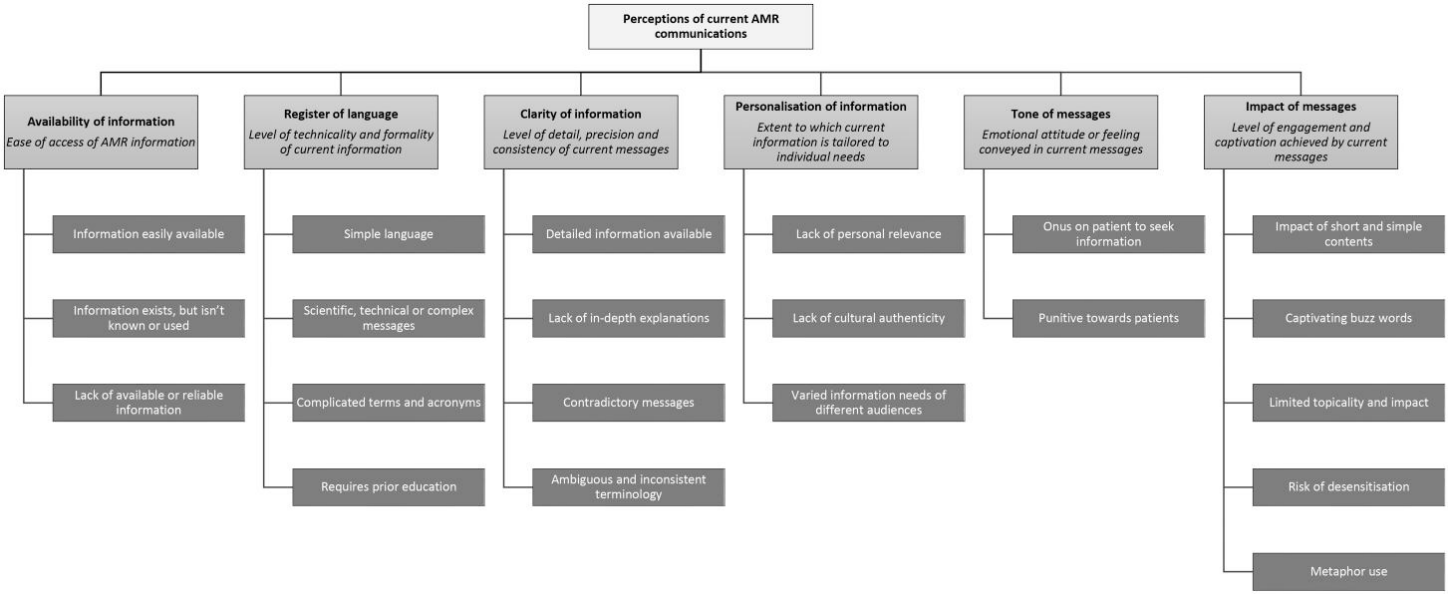
Main qualitative themes and sub-themes identified through reflexive thematic analysis.

Six main themes were identified, each with several sub-themes (see Figure 1). Example quotations for each sub-theme are presented in the Supplementary materials. Participants reflected on any public-facing AMR communication materials. They named examples such as newspaper articles, radio documentaries, webpages of health organisations, social media posts and health campaign leaflets.

### Theme 1: availability of information

The first theme pertained to the ease of accessing AMR-related information. One out of 15 patient participants stated that existing information was readily available, explaining they felt ‘quite lucky because I have an iPad and it has a news feed from every newspaper in the world.’ (P2, patient). Two doctors echoed this sentiment by highlighting available materials suitable for the public from health organisations (e.g. the UK’s Non-tuberculous mycobacterial network), which they shared with patients during consultations.

Some doctors mentioned that informational materials existed that could help with public understanding of AMR but were not well-known or used. They also felt that the design of materials did not always make them suitable to support AMR communication in clinical practice:

‘There is a wealth of material that's been developed, more if you looked online, to talk about public health and also in collaboration with British Society for Antimicrobial Chemotherapy. But the sad thing is those are not readily known outside when you look at the news, radio, TV, social media… none of that comes across.’ (P16, doctor).

‘And there's the Antibiotic Guardian [campaign]… I have to say, on a day-to-day basis… I'm not sure how applicable these are to frontline clinicians, actually.’ (P23, doctor).

Most patients and doctors noted a lack of public-facing AMR information materials. Patients felt that ‘we don’t seem to promote it [the topic of AMR] enough’ (P15, patient) and noted the potential for misinformation ‘because I've sometimes fallen into the trap of, oh, what does Facebook say on my local chat page’ (P9, patient). Doctors were generally unaware of resources for daily practice. One doctor remarked: ‘there's no section in our Trust internet that gives you patient friendly booklets or like patient friendly descriptions of different things’ (P20, doctor).

### Theme 2: register of language

The second theme pertained to the level of technicality or formality in current language used to communicate about AMR. One patient felt that public information about AMR was ‘pretty easy to understand’ (P2, patient). All other patients described the information they had encountered on AMR as complicated, technical and confusing. A more detailed exploration of AMR-related terminology, revealed that all patient participants struggled with the acronym of ‘AMR’ and experienced most of the existing terminology as too scientific.

‘I feel like the words should be in simpler terms so people can get to understand because there's one [newspaper] article I come across… I didn't really understand what was there. … I was trying to read it. So … I had to browse. I had to read up on the article like to see what it was really about, but I still couldn't get it.’ (P5, patient).

Doctor participants shared the patients’ perceptions, arguing that current language, even in public-facing materials, was appropriate for healthcare professionals and experts, but not for lay populations: ‘OK, a lot of them [the terms] are quite clunky. Perhaps difficult for sort of lay people to understand, or I don't know, just it sounds too kind of scientific.’ (P30, doctor)

Patients stated that most materials required a level of literacy, prior education and existing knowledge, in order for them to be understood: ‘You know I didn't do GCSE biology and in my mid-late 40s I've had to re-educate myself about antibiotics’ (P9, patient)

‘I think all of these [AMR-related] words are very relevant but it’s dependent on our interpretation, like how we interpret the wording, this is based entirely on our experiences, our own knowledge, our capability of having infection and diseases so, I guess literacy plays a role like… if your education level is very poor then you are less likely to understand some of the terminology.’ (P15, patient).

### Theme 3: clarity of information

The third theme pertained to the level of detail, accuracy, clarity and consistency in current AMR messages. While one patient and one doctor explicitly stated that they found AMR information to be comprehensive, most others highlighted a lack of clarity.

Patients stated that some information appeared to be very superficial. They gave examples of—in some cases—outdated medical recommendations that were made without clear explanations: ‘And we're always told you should finish the whole course. But no one explains why you should finish the whole course.’ (P2, patient).

This lack of clear and accessible patient facing materials was seen as problematic, as doctors perceived that time was often too limited for them as clinicians to provide patients with detailed explanations, especially in acute settings.

‘I think in terms of specific discussion about how we're going to reduce the risks of antibiotic resistance in your particular case, within each individual patient… Probably isn't done that often at the sharp end.’ (P30, doctor).

Doctors described the contradictory or ‘schizophrenogenic’ (P16, doctor) nature of messaging, where antibiotics are hailed as essential life-savers—for example in sepsis treatment—yet their use is discouraged. Participants also noted that while public information materials warn of dangerous side effects and future resistance, there are simultaneous messages that stress the need to complete a full course, presenting seemingly opposing advice.

The terminology used in public information was perceived as highly ambiguous. Some patients indicated that ‘antibiotic resistance’ may suggest that the antibiotic becomes resistant. ‘Drug-resistant infections’ was criticized by some for being too vague: ‘The only problem I have with drug-resistant infections is, you know, it could be any drug. It could be, I don't know, Aspirin. So, I think it's too vague for me.’ (P5, patient)

Overall, both patient and doctor participants deplored that ‘it [terminology] is used interchangeably and it's confusing at times’ (P16, doctor), leading some to call for a complete overhaul of terminology:

‘I think there needs to be, in my opinion, a new term altogether that covers all of this and relates to all of this and is explained in very, very simple terms, because it could cover a range. Because there's like, you know, different parts to this and … all those important points need to be captured under one term.’ (P6, patient).

### Theme 4: personalisation of information

This theme explored how well public-facing information is delivered in appropriate ways for a diverse public. Both patients and doctors questioned the relevance of current approaches to messaging in terms of their reach and relevance. One patient elaborated on the diversity of backgrounds and individual communication needs:

‘Everyone has a different opinion and different viewpoint, like where they come from and what society and what culture they have been brought up in. How they view illnesses or what other diseases and infections they have had. And prior experiences that they have encountered.’ (P15, patient).

Similarly, one doctor cited BBC Radio 4 documentaries on AMR as insightful but questioned their appeal to younger generations, who primarily get news from social media.

Patients additionally highlighted the lack of cultural authenticity, with one person sharing their difficulties of explaining the concept of resistance to family members from different cultural backgrounds:

‘Being like from South Asian background is just like: an antibiotic should clear it. […] For extended family it was very, very difficult to explain it. My mother-in-law gets it now. But yeah, I think there's not much awareness about it and stuff and it's not really communicated.’ (P6, patient).

Similarly, another patient highlighted that the scientific approach of current communications did not align with their alternative view of medicine that was shaped by culture and family:

‘Coming from a family where we have all had a holistic view of medication, of therapy, and a contemporary approach rather than a scientific one to treat infection and disease, I’ve always been brought up in an environment where we don’t use medication to treat or to prevent cause and are very sceptical of accessing NHS [National Health Service] services as a result.’ (P15, patient).

Doctors also highlighted the need to differentiate AMR risk communication based on specific target audiences. They suggested that mass communication to the public as a whole should discourage antibiotic overuse, whereas more nuanced and targeted messaging would be required for patients, and specifically for those with drug-resistant infections. Further differentiation would be required for acute and chronic patients or asymptomatic carriers of multidrug-resistant organisms.

### Theme 5: tone of message

This theme pertained to emotional undertones and feelings evoked by public-facing AMR health messages. Most patients felt that the onus was on them to pro-actively inform themselves about the topic: ‘Do we have time or energy to research, to inform ourselves? Who are we relying on: culture or family education?’ (P9, patient).

One patient described public messaging as off-putting, ‘draconian’ and discouraging of health-seeking behaviours.

‘From experience, some of the NHS [National Health Service] publications and posters leaflets have been dire. Absolutely dire. […] What I've seen in written communication, it's sort of quite draconian posters, it's like ‘Antibiotics may not be needed in your situation. You may need other medication or none at all’, and, and that's all it says. So that's just one small example, and for me, and for some men who never want to speak to a GP […]. Actually, that's even putting them off even more from contacting [a] health provider.’ (P9, patient).

This view was supported by a doctor who reflected on messaging for an outbreak of a drug-resistant infection in the community, which was perceived as attributing undue blame to patients.

‘People felt that they were made felt responsible for the issue and the fact that they were asking for more antibiotics […] they were saying […] when you're not giving us the information, you're making us feel that we are responsible for what you should be responsible.’ (P16, doctor).

Drawing on their professional experiences communicating about AMR, doctors highlighted importance of a supportive tone or even a positive frame in messaging about AMR and reducing antibiotic use, for example highlighting good news first. One doctor gave the following example: ‘Viruses can be quite unpleasant, but they don't require antibiotics. And that's brilliant news, because what you don't want is me to give you unnecessary antibiotics.’ (P21, doctor)

### Theme 6: impact of message

The final theme covered the extent to which existing messages captivate their audiences. In a few instances, specific messages and terminology were perceived as impactful and effective. One doctor recalled an Instagram video that convinced a friend to finish their antibiotic course. They explained its effectiveness: ‘Very short, very simple. It was all of one sentence and a video clip.’ (P29, doctor). Additionally, many patient and doctor participants were attracted to the catchy ‘buzzword’ (P13, patient) of ‘superbugs’, even though others deemed it inappropriately ‘silly’ (P2, patient), with one person elaborating: ‘Superbug is just something like, I’m a superstar, are you a singer or are you an actor or …[laughs].’ (P13, patient).

However, apart from these isolated examples, most participants stated that public-facing information lacked interest, topicality and impact: ‘It's not… a topical thing that you would immediately pick up and start reading.’ (P2, patient)

‘There are classic adverts that have got through to people like the AIDS advert where they have the tombstone and there were a lot of comments on that and possibly with the benefit of hindsight it was politically incorrect, but it worked at the time. It made people wake up. It’s that sort of punch that you need for people that aren’t informed.’ (P13, patient).

One doctor additionally warned about the risk of de-sensitisation, explaining that ‘when you bombard people with messages and information, you get numb’ (P16, doctor).

To improve engagement and impact of messages, some doctors described the use of metaphors, mostly drawing on the domain of war and fighting: ‘I do keep talking in clinic about keeping my powder dry.’ (P24, doctor)

‘Sometimes I use the word like ‘this gun is not effective so we have to use a more strong gun to kill the bug and because the gun is like powerful’. So there is a collateral damage, it is expected. So, in case of like damage to the other organs, like close monitoring and more frequent blood tests are required.’ (P25, doctor).

## Discussion

Findings from focus groups with UK patients and hospital doctors consistently indicated that current AMR messaging is ineffective. With a few exceptions, information was perceived as difficult to access, overly scientific in register, unclear or confusing, personally irrelevant, punitive in tone, and limited in overall ‘punch’ and impact. Independent of the qualitative results, some patients identified through hospital records and invited to the study contacted the research team to obtain clarification, unaware they had experienced a resistant infection. This in itself is a strong indicator for insufficient communication.

Some of our results align with previous literature. The perceived lack of available information echoes media analyses suggesting that AMR is often side-lined in favour of more relatable news items.^8^ Perceptions that information is complex and abstract, link in with previous experimental findings highlighting the problematic use of scientific acronyms.^23,24^ Participant observations highlighting the contradictory nature of AMR communications align with previous discussions around seemingly competing agendas of sepsis and AMR campaigns.^9^ Perceived lack of personally relevant narratives reinforces earlier calls to emphasize individual susceptibility.^22^ Finally, participants’ sense that message wording lacked impact aligns with suggestions that evoking threat may create stronger impressions on lay audiences.^19,20^

However, our qualitative results also revealed some novel themes, extending the largely quantitative data from previous work. Findings regarding the lack of cultural authenticity and perceptions around ‘draconian’ language offer worrying insights into the need for more nuanced and empathetic messaging.

### Interpreting findings with the ELM

**Figure 2.**
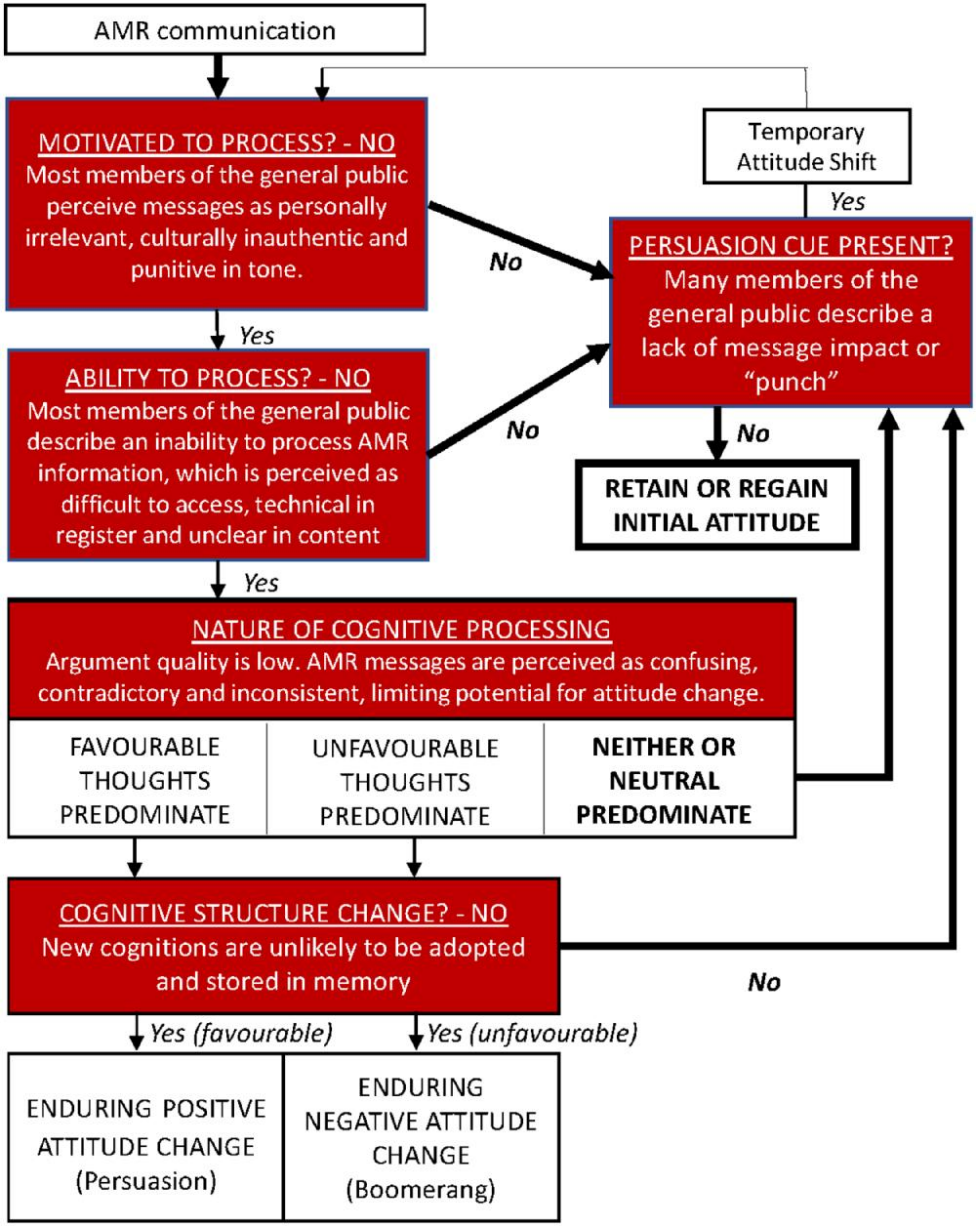
Mapping the present findings against the ELM. This figure is an adaptation of the model’s original schematic overview.^25^ Components shaded (in red) highlight processing barriers identified in the current study. Dominant processing pathways are highlighted by bolded arrows. The most likely outcome of AMR messaging (‘retain or regain initial attitude’) is emphasized using a bold frame.

Mapping our findings against the ELM provides a theory-based understanding of current weaknesses and suggests possible message improvements (see Figure 2). The model maintains that motivation and capability are key criteria that determine the level of central route processing and elaboration.^25,33,34^

In AMR communication, motivation may be hindered by problematic message personalisation and tone. Indeed, our focus groups indicated that current information often felt culturally or personally irrelevant. Participants also noted that current campaigns seemed to unfairly blame or reprimand the public for antibiotic overuse, which could further discourage engagement.

Our research also questioned the public’s ability to engage deeply with AMR messages. The first three themes—information availability, register, and clarity—highlighted key barriers to processing. If people have limited access to clear and accessible information, deeper engagement is unlikely.

A final factor limiting central processing is poor argument quality, with participants describing perceptions of contradictory messages. Examples included calls to reduce antibiotic use, while urging immediate use for suspected sepsis and stressing course completion. Interestingly, participants appeared unaware of updated guidance to take antibiotics as directed, which in itself indicates insufficient communication. These mixed messages reinforce peripheral processing, hindering lasting attitude change.

According to the ELM, peripheral processing can lead to attitude change, but changes are less stable over time. Given the lower levels of elaboration, peripheral processing is influenced by simple heuristics, biases, and superficial cues, such as judgements of the message source, the recipient’s mood, and the matching of message content to audience characteristics.^33^ This means it is essential to consider emotional resonance and overall message impact when designing communications. Our focus groups identified barriers to this peripheral impact in AMR messages, which were seen as lacking the emotional gravitas of previous health campaigns. The term ‘superbugs’ sparked mixed reactions—some found it catchy and meaningful, while others saw it as silly or misleading. Our results suggest that peripheral processing of AMR messages is unlikely to change attitudes and beliefs.

### Improving AMR communications and future research directions

Based on the combined insights from previous research, the present qualitative data and theoretical insights of the ELM, we propose the following recommendations to improve public AMR communications. Recommendations are mapped against the key ELM components.

#### Promote motivation to engage

Tailoring messages to target audiences by emphasising personal relevance is crucial. Insights from our focus groups suggest that adapting messages to individuals’ existing experiences and beliefs may be effective in making contents more appropriate and aligning them with community needs. This echoes previous work by the Wellcome Trust, which highlighted the importance of emphasising personal susceptibility and the relevance of AMR to present-day decision-making.^22^ Similarly, findings from a review of AMR awareness in the World Health Organisation African region denotes the importance of tailoring messages in low and middle income countries where context-specific determinants have a great potential to close existing knowledge gaps.^35^ Inspiring initiatives are underway, which aim to communicate AMR patient stories in an attempt to connect with audiences,^36,37^ but further research across diverse cultural settings may be needed to adapt AMR messages to different belief systems. The message tone also plays a critical role in fostering engagement. Our focus group findings revealed deterring effects of punitive language. This is consistent with prior literature emphasising the need for positive, solution-focused contents.^19,22^ Finally, while this study did not examine participants’ sense of personal impact on AMR, the success of recent pledge-based campaigns suggests that incorporating messages around self-efficacy and goal-setting could strengthen motivation and warrants further exploration.^38^

#### Improve ability to process

Access to jargon-free AMR communications is essential. Our focus groups suggest that social media could be leveraged for easier distribution of simple information. This supports past findings that using multiple channels, including non-traditional media such as social media platforms targeting different age groups, is most effective. Reducing technical language is crucial to making messages easier to process, echoing previous literature that discouraged the use of ‘AMR’ and ‘antimicrobial resistance’ in public communications.^23,24,39,40^ Additional considerations, which extend the scope of the present English-language research, may involve meaningful translation of key terminology to bridge linguistic barriers.^41^

#### Strengthen argument quality to convince

To promote attitude change when people are motivated and able to elaborate on a message, it is important to improve argument quality by reducing inconsistencies and contradictions. Clearly presented information about the ineffectiveness of antibiotics against viruses has been proven to be effective.^17^ Similarly, fully explaining the rationale behind confusing advice—such as the need to complete a course of antibiotics—could improve public understanding and resolve ambivalence. At a broader policy level, it is crucial to align seemingly competing campaigns targeting sepsis and AMR^9^ and increase consistency of health advice across stakeholders and organisations.

#### Maximize impact of peripheral cues

To facilitate attitude change through peripheral processing, it is essential to harness superficial message cues. Focus groups highlighted the effectiveness of simple visuals encountered in a social media post, which echoes previous findings from education campaigns regarding the importance of visual information salience.^15,16^ Fear-based elements could be incorporated for impact, but care should be taken not to desensitize the audiences.^19^ Indeed, insights from the climate change discourse favour messaging approaches that align with people’s reference points and increase message salience by targeting contents to existing beliefs and everyday concerns.^42^ Additionally, buzzwords like ‘superbugs’ could be used, although different connotations should be carefully considered. A novel tool for both central and peripheral engagement may be metaphors, which are likely to provide emotional impact, captivate audiences and thereby increase memorability of information.^43^ While some focus group doctors already reported using metaphors to increase patient engagement, future research is required to develop novel figures of speech that offer alternatives and go beyond conventional war narratives to construct meaning.^14^

### Study limitations

The present study has several limitations. As with most qualitative studies, findings may not be generalisable. Despite purposive sampling, most doctors came from a single English hospital trust. While doctors worked across different departments, it is possible that clustering effects of hospital culture played a role, thereby introducing bias.

Furthermore, the study’s focus on public-facing AMR communications was comparatively broad. Focus group participants discussed various types of AMR communications, referencing mostly public-facing but sometimes also patient information, occasionally failing to distinguish between them. As a consequence, our results may partially reflect clinical insights. Nonetheless, such input still highlights the complexity of AMR communication.

Given our sampling strategy that focused on individuals with experiences of drug-resistant infections, most participants reflected on this particular aspect of AMR rather than its broader impact on modern healthcare (e.g. implications for routine surgeries or cancer treatment). Future work may be required to assess AMR communications specifically pertaining to the threat for vital modern medical technologies.

A final limitation pertains to our chosen theoretical framework. While recognized as a leading model,^26^ the ELM has faced criticism for being largely descriptive and of uncertain relevance to modern communication settings. Its assumptions, particularly the binary processing routes, have been challenged, with some evidence suggesting that both routes can operate simultaneously.^44^ We would argue that every theoretical framework has shortcomings and have chosen the ELM due to its apparent relevance for interpreting our data and superior fit compared with alternative models.

### Conclusions

Public AMR communication needs a theory-driven, evidence-based reset. Our findings show current messages are often inaccessible, technical, unclear, or irrelevant—hindering engagement and lasting impact, as explained by the ELM. To improve, we should: (1) boost motivation through personal relevance and positive framing, (2) enhance understanding by simplifying language, (3) increase message clarity and consistency, and (4) use engaging cues like emotion, visuals, and metaphors to strengthen impact. Novel, multichannel campaigns that integrate these elements, as well as lessons from past efforts, could advance meaningful public engagement.

## Supplementary Material

dlaf148_Supplementary_Data

## Data Availability

Example quotations for each theme are provided in the supplementary materials. Due to the potentially identifiable nature of some participant statements within the qualitative transcripts (e.g. making references to specific cultural upbringings or geographic contexts), full texts are not shared publicly, but may be obtained from the authors upon reasonable request.

## Supplementary data

Table S1 is available as Supplementary data at *JAC-AMR* Online.
