# Supplementary material for: Why antimicrobial resistance messaging fails: qualitative insights interpreted through the elaboration likelihood model
Source: JAC Antimicrob Resist. 2025 Aug 8;7(4):dlaf148. doi: 10.1093/jacamr/dlaf148 (PMC12342773; doi:10.1093/jacamr/dlaf148)
Supplement: dlaf148_Supplementary_Data [file dlaf148_supplementary_data.docx]

**Why Antimicrobial Resistance Messaging Fails: Qualitative Insights Interpreted Through the Elaboration Likelihood Model**

**Supplementary Materials**

**Focus group topic guide for patient participants**

*Note: The topic guide contained additional questions to explore broader conceptualisations and beliefs related to AMR, which will be reported in a separate publication.*

QUESTIONS ABOUT PATIENT EXPERIENCE

1. Can you briefly tell me about your personal experience with infection?

*Probes:*

- *When you had an infection recently, were you aware that it was an infection that was resistant to some antibiotics? What were you told about this, and by whom?*
- *How did antibiotic resistance affect your recovery?*

1. How did you communicate your experience of infection and antibiotic resistance to friends and family?

Probes:

- *What language did you use to share your experience?*
- *What challenges (if any) did you encounter in telling others about having a resistant infection?*

CONCEPTUALISATION OF ANTIBIOTIC RESISTANCE

Next, we are going to discuss your opinions about antibiotic resistance and about current risk communication on the topic. Antibiotic resistance happens when bacteria change over time so that they no longer respond to antibiotic medication. This means that bacterial infections like pneumonia, urinary tract infections or meningitis may no longer be treatable with existing antibiotic drugs.

1. Thinking about your recent experience of an infection, what does antibiotic resistance mean to you?

*Probes:*

- *What do you think are the implications for you personally?*
- *How does antibiotic resistance affect people?*

*What do you see as the implications of antibiotic resistance for society? For future generations?*

PERCEPTIONS OF CURRENT RISK COMMUNICATION ABOUT ANTIBIOTIC RESISTANCE

1. Have you come across any written or spoken information about antibiotic resistance? This may include newspaper articles, online information, flyers, leaflets, TV or radio news reports or verbal information received by healthcare staff.
2. What do you think of the information and risk communication about antibiotic resistance that you are aware of?

Probes:

- *How effective is existing information in making people aware of the risk of antibiotic resistance?*
- *How easy is it to understand existing information about antibiotic resistance?*

1. There are also several public misunderstandings associated with antibiotics and antibiotic resistance. One misbelief is that antibiotics can be used to treat viral illnesses such as colds or flu. This is incorrect, because antibiotics are only effective for treating bacterial infections. If people incorrectly believe antibiotics can cure colds, they might end up expecting or demanding antibiotic medicines when they aren’t needed. This could lead to an overuse of antibiotics and an increase of antibiotic resistance.

What do you think causes this common misunderstanding?

*Probes:*

- In your opinion, what do people know about the difference between viruses and bacteria?
- How might previous experiences shape people’s beliefs (e.g., previous use of antibiotics for viral illnesses)?

1. Another misbelief is that antibiotic resistance is the result of the body becoming resistant to antibiotics. This is incorrect because antibiotic resistance involves bacteria becoming resistant. This misunderstanding is problematic because it may mislead people to think that antibiotic resistance will not affect them personally if they—as an individual—do not use antibiotics. As a result, they might fail to understand the risks associated with contracting resistant bacteria from other people. People with this belief are likely to underestimate the severity of antibiotic resistance risks and misjudge their own susceptibility.

What do you think causes this common misunderstanding?

*Probes:*

- In your opinion, how do people think about risks of spreading antibiotic resistance?
- To what extent does people’s knowledge about other medical conditions (e.g., drug dependence and addiction) shape beliefs about antibiotic resistance?

1. When communicating about antibiotic resistance, media and health organisations have been using several different names interchangeably. Example names are antibiotic resistance, antimicrobial resistance, AMR, bacterial resistance, drug-resistant infections and superbugs. In your opinion, how effective are the existing names?
2. In your opinion, what are advantages and disadvantages of the different names? Which of the existing terms do you think is most useful?

*Probes:*

- *What are advantages and disadvantages of “antibiotic resistance”?*
- *What are advantages and disadvantages of “antimicrobial resistance”?*
- *What are advantages and disadvantages of “AMR”?*
- *What are advantages and disadvantages of “bacterial resistance”?*
- *What are advantages and disadvantages of “drug-resistant infections”?*
- *What are advantages and disadvantages of “superbugs”?*

**Focus group topic guide for doctor participants**

QUESTIONS ABOUT PROFESSIONAL EXPERIENCE IN TREATING AND COMMUNICATING RESISTANCE

1. Can you briefly tell me about your professional experience with treating severe infection caused by multidrug-resistant organisms?

*Probe:*

- *How did* *antibiotic resistance affect your treatment choices and patient outcomes?*

1. How do you communicate information about antibiotic resistance to patients or fellow doctors?

*Probes:*

- *What language do you use?*
- *What challenges (if any) do you encounter in communicating about antibiotic resistance?*

PERCEPTIONS OF CURRENT RISK COMMUNICATION ABOUT ANTIBIOTIC RESISTANCE

1. What do you think of existing risk communication materials about antibiotic resistance for lay audiences?

Probes:

- *How effective is existing information in making people aware of the risk of antibiotic resistance?*
- *How easy is it to understand existing information about antibiotic resistance?*

1. When communicating about antibiotic resistance, media and health organisations have been using several different names interchangeably. Example names are antibiotic resistance, antimicrobial resistance, AMR, bacterial resistance, drug-resistant infections and superbugs. In your opinion, how effective are the existing names?

*Probe:*

- *Which of the existing terms do you think is most useful and why?*

**Table 1**

***Illustrative Quotations for All Sub-Themes Identified in the Reflexive Thematic Analysis***

| **Themes** | **Illustrative quotes** |
| --- | --- |
| **Availability of information** | |
| Information easily available | *I'm* *quite lucky because I have an iPad and it has a news feed from every newspaper in the world. You know, I can read lots of different articles. I don't think anything was difficult to understand.* (P2, Patient) |
| Information exists but isn’t known or used | *There is a wealth of material that's been developed. More if you looked online. To talk about public health and also in collaboration with British Society for Antimicrobial Chemotherapy. But the sad thing is those are not readily known outside when you look at the news. Radio, TV, social media, none of that comes across.* (P16, doctor) |
| Lack of available or reliable information | *Well, it's about that lack of information and the lack of like campaigns and you know, information that's shared and that there should be a lot more… there should be like a very strong campaign or more real authentic information on social media and so forth because there isn't anything* (P6, patient) |
| **Register of language** | |
| Simple language | *There is patient information on that website for patients […] And it's got the basics and beyond the basics. So for your, I mean, I jokingly say to people we've got two I could share with you: One is for the Sun reader and the other is for the Times reader, and they've got different reading ages.* (P21, doctor) |
| Scientific, technical or complex messages | *Some articles, like whatever it says, it's not really explicit, so I feel like the words should be in simpler terms so people can get to understand because there's one article I come across, I didn't really understand what was there.* (P5, patient) |
| Complicated terms and acronyms | *I think that the only thing familiar is antibiotics, like amoxicillin, that’s the only [term]. I’m not very good at medical terms myself.* (P14, patient) |
| Requires prior education | *I guess literacy plays a role like… if your education level is very poor then you are less likely to understand some of the terminology.* (P15, patient) |
| **Clarity of information** | |
| Detailed information available | *One exemplary communication initiative and leaflet and I think booklet as well has been written as part of the NTM standards of care work. […] It allows you to share really quite detailed information with your patients, who are often highly educated patients of middle age or sort of approaching pension age and they do have a lot of questions.* (P24, doctor) |
| Lack of in-depth explanations | *I came across a lot of materials about antibiotics in the system, and I couldn't really understand it in clarity, so now [I’m] little bit confused.* (P1, patient) |
| Contradictory messages | *We give people these schizophrenogenic messages that, you know, bacteria kill. You need antibiotics. But at the same time, you know, don’t take antibiotics, bacteria are good. So I don’t really know how to strike a balance there because both are true.* (P16, doctors) |
| Ambiguous and inconsistent terminology | *I think that the term antibiotic resistant it leads you to believe that it’s the antibiotic [that becomes resistant]* (P2, patient) |
| **Personalisation of information** | |
| Lack of personal relevance | *Everyone has a different opinion and different viewpoint, like where they come from and what society and what culture they have been brought up in. How they view illnesses or what other diseases and infections they have had. And prior experiences that they have encountered.* (P15, patient) |
| Lack of cultural authenticity | *I think culturally it there is not enough information. […] There's nothing much really for all communities to like really understand what's going on.* (P6, patient) |
| Varied information needs of different audiences | *I think we're talking about two different sorts of communication or circumstances of communication here. One is when the patient* *needs really complex antibiotics or antibiotics regimes and we're trying to explain, they got the resistance. The other is the sort of stewardship angle of explaining either they don't need antibiotics or they need a very simple antibiotic* (P17, doctor) |
| **Tone of messages** | |
| Onus on patient to seek information | *For me, I don’t think I have come across anything like that [AMR information] or I’ve not had the interest to actually go do research on that, actually, not really.* (P10, patient) |
| Punitive towards patients | *From experience, some of the NHS publications and posters leaflets have been dire. Absolutely dire. […] What I’ve seen in written communication, it’s sort of quite draconian posters, it’s like antibiotics may not be needed in your situation. You may need other medication or none at all, and, and that’s all it says. So that’s just one small example, and for me, and for some men who never want to speak to GP or woman. Actually, that’s even putting them off even more from contacting health provider.* (P9, patient) |
| **Impact of messages** | |
| Impact of short and simple contents | *The one almost sort of lay facing piece of media I have come across that is on Instagram […] It was very short, very simple. It was all of one sentence and a video clip*. (P29, doctor) |
| Captivating buzz words | *I think the ‘Superbug’ one, you know, is perhaps the one that might have most sort of traction, but that's got sort of some of the drama around needing antibiotics because the superbugs are around* (P17, doctor) |
| Limited topicality and impact | *But I've seen like posters in my GP surgery about “do you really need antibiotics” - big question mark. I'm talking about resistance, and I remember vaguely a news item on TV talking about it in like a two-minute slot, but nothing that made an impact.* (P4, patient) |
| Risk of desensitisation | *When you bombard people with messages and information, you get numb. It's like we know what you get too much of the same, we shut down.* (P16, doctor) |
| Metaphor use | *“Sometimes I use the word like this gun is not effective so we have to use a more strong gun to, to kill the bug and because the gun is like powerful. So there is a collateral damage, it is expected. So, in case of like damage to the other organs, like close monitoring and more frequent blood tests are required”* (P25, doctor) |
